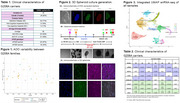# Transcriptomic analysis from 3D cultures of Presilinin‐1 G206A carriers reveals genetic expression differences associated with age‐of‐onset variability

**DOI:** 10.1002/alz70855_104923

**Published:** 2025-12-24

**Authors:** Katrina Celis, Anthony J Griswold, Farid Rajabli, Aura M Ramirez, Younji Nam, Larry D Adams, Patrice G Whitehead, Pedro R Mena, Jose J. Sanchez, Charles G. Golightly, Brooke A. DeRosa, Concepcion Silva‐Vergara, Heriberto Acosta, Katalina F. McInerney, Derek M. Dykxhoorn, Briseida E. Feliciano‐Astacio, Michael L Cuccaro, Jeffery M Vance, Margaret Pericak‐Vance, Juan I Young

**Affiliations:** ^1^ John P. Hussman Institute for Human Genomics, University of Miami Miller School of Medicine, Miami, FL, USA; ^2^ University of Miami Miller School of Medicine, Miami, FL, USA; ^3^ Universidad Central del Caribe, Bayamón, PR, USA; ^4^ Clinica de la Memoria, San Juan, PR, USA; ^5^ Dr. John T. Macdonald Foundation Department of Human Genetics, University of Miami Miller School of Medicine, Miami, FL, USA

## Abstract

**Background:**

The Presenilin‐1 (*PSEN1*) G206A mutation has been identified in Puerto Ricans (PRs) with Alzheimer disease (AD), as a founder effect in the African ancestral background. The G206A mutation exhibits large age‐of‐onset (AOO) variation (range 30‐90 years) between carriers. Genetic markers associated with AOO variability have been reported from association studies, but the underlying mechanism is unknown. Here, we used induced pluripotent stem cells (iPSCs) derived 3D cultures from G206A carriers to evaluate cell type transcriptomic differences associated with AOO variability.

**Method:**

We reprogrammed six iPSCs lines from AD *PSEN1* G206A carriers, three early (<50 years) and three late (>60 years) AOO. Those iPSCs were used to generate spheroid 3D cultures containing neurons, astrocytes, OPC, oligodendrocytes and endothelial cells. Cells were collected at day 75 of differentiation for single nucleus RNA sequencing using the10X Genomics platform and data was analyzed for transcriptomic differences between early and late AOO, using Seurat software and adjusted *p*‐value <0.05 as significance cut‐off.

**Result:**

We sequenced a total of 38,577 nuclei and detected an average of 2,700 genes per cell. We identified eight cell type clusters. The oligodendrocyte cluster showed the highest expression of *PSEN1*. No significant difference was observed for of *PSEN1* or its substrates in any cluster between early and late AOO groups. However, we found significantly decreased expression of genes involved in presynaptic differentiation (*LRFN5*), ER stress response and apoptosis (*RNF13*), neuroinflammation (*FUT8*) and lipid metabolism (*SOX9‐AS1)* in early vs late AOO in multiple cell type clusters. Additionally, we found significantly higher expression of genes involved in vascular (*CAV1, COL4A1*), neurodevelopment and lipid metabolism (*PSAP*) in early vs late AOO carriers.

**Conclusion:**

Our analysis identified transcriptomic changes between spheroid 3D cultures of early and late AOO G206A carriers in genes previously implicated in multiple studies of AD pathogenesis and neurodevelopmental disorders. Our results suggest that pathways involved in vascular morphogenesis, lipid processing, protein aggregation and neuronal regulation may be contributing to the variability in AD AOO observed in G206A carriers.